# What Affects Attendance and Engagement in a Parenting Program in South Africa?

**DOI:** 10.1007/s11121-018-0941-2

**Published:** 2018-08-18

**Authors:** Yulia Shenderovich, Manuel Eisner, Lucie Cluver, Jenny Doubt, McKenzie Berezin, Sybil Majokweni, Aja Louise Murray

**Affiliations:** 1 Institute of Criminology, University of Cambridge, Sidgwick Avenue, Cambridge, CB3 9DA England; 2 Department of Social Policy and Intervention, University of Oxford, Barnett House, 32 Wellington Square, Oxford, OX1 2ER England; 3 Department of Applied Psychology, New York University, 246 Greene Street, New York, NY 10003 USA; 4 HIV Mental Health Research Unit, Department of Psychiatry, University of Cape Town, Cape Town, South Africa

**Keywords:** Parenting, Child maltreatment, Adolescents, South Africa

## Abstract

Parenting programs are a promising approach to improving family well-being. For families to benefit, programs need to be able to engage families actively in the interventions. Studies in high-income countries show varying results regarding whether more disadvantaged families are equally engaged in parenting interventions. In low- and middle-income countries (LMICs), almost nothing is known about the patterns of participation in parent training. This paper examines group session attendance and engagement data from 270 high-risk families enrolled in the intervention arm of a cluster-randomized controlled trial in South Africa. The trial evaluated a 14-week parenting intervention aiming to improve parenting and reduce maltreatment by caregivers. The intervention was delivered in 20 groups, one per study cluster, with 8 to 16 families each. Overall, caregivers attended 50% of group sessions and children, 64%. Using linear multilevel models with Kenward-Roger correction, we examined child and caregiver baseline characteristics as predictors of their attendance and engagement in the group sessions. Variables examined as predictors included measures of economic, educational, and social and health barriers and resources, as well as family problems and sociodemographic characteristics. Overall, the study yielded no evidence that the level of stressors, such as poverty, was related to attendance and engagement. Notably, children from overcrowded households attended on average 1.2 more sessions than their peers. Our findings suggest it is possible to engage highly disadvantaged families that face multiple challenges in parenting interventions in LMICs. However, some barriers such as scheduling, and alcohol and substance use, remain relevant.

**Electronic supplementary material:**

The online version of this article (10.1007/s11121-018-0941-2) contains supplementary material, which is available to authorized users.

Child maltreatment is common and costly, and it disproportionately affects low- and middle-income countries (LMICs) (Ward et al. 2016). Recently, prevention and reduction of physical and emotional child maltreatment has become more prominent in the global agenda (WHO 2016). The new Sustainable Development Goals include ending all forms of violence against children. Responsive and consistent parenting has been identified as a protective factor that promotes children’s health and development in low-income and high-stress contexts (Murphy et al. 2017). Therefore, there is a need to build knowledge on promoting effective parenting in LMICs. Parenting interventions are a promising approach to improve parenting, and to reduce and prevent child maltreatment (e.g., Mikton and Butchart 2009; Barlow et al. 2013). They can also target other outcomes, such as parent mental health, child externalizing behavior, and substance use (e.g., Chen and Chan 2016). These interventions include a range of designs, and are usually delivered individually or in groups over several weeks, based on a treatment manual.

Group parenting interventions rely on participants attending sessions and engaging with the content. Several studies demonstrated that the extent of participation is important in determining benefits gained from a parenting intervention. The levels of participant attendance and engagement in sessions were linked to intervention outcomes in several studies in the United States (US) (Baydar et al. 2003; Gross et al. 2009). Other US studies have found that engagement in sessions—but not attendance—predicted outcomes (Garvey et al. 2006; Nix et al. 2009). A recent Dutch study found that more sessions attended by parents predicted better parenting behavior but not child behavior (Weeland et al. 2017).

A key motivation for the current study, informed by frameworks such as the family stress theory, is that families with multiple stressors are at a higher risk for strained family relationships (Smith et al. 2016). Yet the families who experience multiple stressors and competing demands on their time may be the least likely to attend parenting sessions. For instance, families with limited social support may have access to fewer alternative caregivers who can look after other children in the household or care for ill family members during sessions (Farrelly and McLennan 2009). We consider the predictors relevant for child maltreatment and harsh parenting (e.g., see Meinck et al. 2017), the key outcomes of the current intervention. Drawing on categories in previous research, we look at four groups of predictors: economic and educational barriers and resources, social and health barriers and resources, parenting and child behavior, and sociodemographic factors.

Among the economic and educational factors, socio-economic status is perhaps the most commonly examined predictor of participation in parenting interventions. A few studies have demonstrated lower attendance in families with lower socio-economic standing (e.g., Peters et al. 2005), while others did not find such an effect (e.g., Nix et al. 2009). The discrepancy is perhaps in part due to a mix of universal and high-risk samples, different interventions, study contexts, and methods of capturing the socio-economic situations of families. Studies have examined indicators such as family income (Haggerty et al. 2002), caregiver education (Nix et al. 2009), and family occupational prestige (Kazdin 2000). In LMICs such as South Africa, overcrowded household is another relevant indicator of family disadvantage (Meinck et al. 2017). Alternative measures may have different effects on participation. Many programs provide childcare, refreshments, and transport to ensure that the families with an economic disadvantage can participate. However, the effects of limited educational experience may be harder to address (Eisner and Meidert 2011).

Social and health barriers studied in parenting interventions include parental depression, substance use, and social support (Morawska et al. 2014). Research has found significant relationships between caregiver depression, parenting stress, and dropout (Calam et al. 2002). However, results are mixed in terms of whether mental health affects participation, with some research identifying equal or higher engagement in the families with more mental health problems (e.g., Baydar et al. 2003; Smith et al. 2016). We also examine additional measures of mental and physical well-being shown in South Africa to predict child maltreatment, such as caregiver exposure to intimate partner violence, childhood maltreatment, and HIV status (Meinck et al. 2017).

Examining the effect of pre-intervention parenting and child behavior, we may expect that families with greater difficulties find it more difficult to engage. On the other hand, according to the Health Belief Model, parents and children are more likely to participate if they perceive their family problems to be more serious. Indeed, some studies identified perceived family challenges as empirically significant predictors of higher parental participation (Baydar et al., 2016; Gorman-Smith et al. 2002). However, other studies found no relation (Eisner and Meidert 2011; Salari and Filus 2017). Finally, findings are also mixed on the impact of sociodemographic characteristics, such as child age and gender.

Most interventions described in the literature targeted parents of young children and delivered training to caregivers only. As a result, predictors of child participation have been rarely examined in parenting research. However, increasingly, many programs for older children also include sessions for the young people, and studies have shown that child involvement can boost parental engagement (Fleming et al. 2015) and lead to more sustainable changes in the family. Therefore, we also examine predictors of child attendance using analogous predictors as for the caregivers. Lastly, there is little information on what may affect participation in LMICs. The findings on predictors of participation thus far primarily originate from interventions focusing on disruptive child behavior in HICs. One study examined a multicomponent program for caregivers of malnourished children in the Dominican Republic (Farrelly and McLennan 2009). The program focused on nutrition, but also included sessions on child behavior management. The researchers found that none of the eight hypothesized variables predicted attendance.

In summary, research suggests a range of potential predictors affecting participation in parenting programs. In this exploratory study, we assess the effects of relative disadvantage on program participation in a LMIC setting. Given the inconsistent findings in previous studies and paucity of research with adolescents, we did not hypothesize specific predictor effects. This paper aims to contribute to the emerging evidence base by describing attendance and engagement in a parenting intervention among a high-risk sample in South Africa, and by examining the factors associated with the variation in attendance and engagement.

## Methods

### Study Setting

This study was nested within a cluster-randomized trial in the Eastern Cape Province, South Africa, that took place between April 2015 and August 2016. The trial enrolled 552 families in 32 rural and 8 peri-urban clusters, all in communities with high rates of poverty and unemployment. Half of the clusters (16 rural and 4 peri-urban) were randomized to each trial arm. A detailed description is available in the protocol (Cluver et al. 2016b). In short, the treatment arm received the Sinovuyo Teen parenting program, which aims to reduce physical and emotional maltreatment of children and improve parenting. The control arm received a 1-day hygiene information intervention.

### Study Sample

In this study, each participating family enrolled one child and their primary caregiver, defined as the person mainly responsible for the child and residing in the same household at least four nights a week. The caregivers enrolled in the study were primarily mothers and grandmothers, and 3% of the caregivers were male. In the intervention arm, the children were 56% boys, aged 10 to 18 (*M* = 13.7, SD = 2.3). Although the recruitment focused on disadvantaged families, there was substantial variation in the predictors (descriptive information available online). The participants were recruited into the study through door-to-door recruitment and referrals from local community members, schools, and social workers. Recruitment focused on families that already experience conflicts and stress. To be enrolled in the study, families had to reply affirmatively to one of the screening questions on whether there are conflicts between the caregiver and adolescent in the household, and complete the two rounds of baseline assessments. All responses were kept confidential, except in cases of participants requesting assistance, or at risk of significant harm, such as children with recent suicide attempts. Families did not receive monetary incentives for participation, but were given small packs with snacks, stationery, and toiletries to thank them for participating.

### Intervention Characteristics

Sinovuyo Teen is a 14-week manualized program based on social learning theory. The program was developed based on principles from existing research, such as modeling positive behavior and collaborative problem solving. During the development and piloting stages, the intervention was modified for the South African context (Cluver et al. 2016a, c). The intervention consisted of weekly group sessions (four separate and ten joint sessions for caregivers and children) and weekly home practice. Group sessions took place in a community location, such as a community hall or a school. Intervention groups included between 8 and 16 families, with 14 on average. The sessions lasted, on average, 1.8 h and were usually led by two facilitators. In addition, participants who were unable to attend a session received a brief home visit from the facilitators with a summary of the week’s content. Sessions were facilitated by community members and social workers, trained by Clowns Without Borders South Africa, a local non-governmental organization. Facilitators received an initial 5-day training and ongoing weekly supervision and further training.

### Instruments and Measures

#### Attendance and Engagement Outcomes

The number of group sessions attended by children and caregivers in the intervention arm and their average engagement in the sessions were the primary outcomes of interest in this exploratory analysis. Both measures were collected through observations by the research team. Additionally, attendance was cross-checked with facilitator-recorded data. The research team observed 277 sessions, out of the total 279, and 32% of the sessions were double-coded by two observers. Fifteen local Research Assistants were involved in observations after a training in sensitive data collection, as well as in observational research. To measure the level of engagement in sessions, we used a behaviorally anchored 3-point scale (child or caregiver: 1, is quiet or distracted most of the time; 2, participated in parts of the session; 3, participated through most of the session).

#### Baseline Predictors

Baseline interviews were conducted by local Research Assistants and took place at participant homes or other venues, such as schools. All questionnaires were locally piloted in Xhosa. Tablets were used to administer questionnaires to participants. Below, we provide a summary of the baseline measures used as prospective predictors of attendance and engagement. We use similar, but not identical, baseline variables to predict child and caregiver outcomes. For instance, we use child report of parenting to predict children’s participation, and caregiver report to predict caregiver participation. Similar to previous studies (e.g., De Los Reyes and Kazdin 2005), we find low correlations between the child and caregiver reports of the same constructs (between 0 and.24). Where possible, we use predictor and outcome information from the same informant as their perception is more likely to affect their own behavior.

#### Economic and Education Barriers and Resources

*Family poverty* was measured by the Basic Necessities Scale, asking how many household necessities for children families could afford (Pillay et al. 2006). Cronbach’s *α* for this scale was 0.72 (8 items). *Overcrowding* was defined as having more than three people residing per room, per United Nations Humans Settlements Program definition. *Caregiver education* was a dichotomous indicator of whether the caregiver completed primary school.

#### Social and Health Resources and Barriers

As a measure of *caregiver depression*, we included the Centre for Epidemiological Studies Depression Scale, used previously with South African populations (Pretorius 1991). Cronbach’s *α* for this scale was 0.86 (19 items). *Child depression* was measured by the Child Depression Inventory short form (Kovacs 1992), with Cronbach’s *α* 0.64 (10 items). *Child and caregiver HIV* was assessed with the Verbal Autopsy Symptom Checklist (Lopman et al. 2006; Hosegood et al. 2004) and 6 specific items on HIV testing, ARV treatment and CD4 count. HIV status was determined with a conservative threshold of ≥ 3 AIDS-defining illnesses, or self-identification of HIV-positive status, or caregiver report of child status, as children may be unaware of their status. *Alcohol and drug use* among children was measured using two adapted items from the Alcohol and Other Drug Use Module developed by the World Health Organization for the Global School-Based Health Survey. *Alcohol and drug use* among caregivers was measured by four items developed by the research team to assess alcohol and drug use in the past month*. Caregiver social support* was measured with the Medical Outcome Study Social Support Survey (Sherbourne and Stewart 1991), Cronbach’s *α* 0.95 (19 items). *Caregiver experience of intimate partner violence* was measured using a simplified version of the Revised Conflict Tactics Scale Short Form, Cronbach’s *α* 0.85 (6 items). *Caregiver history of maltreatment* was assessed using an adapted version of the ISPCAN Child Abuse Screening Tool-Retrospective, measuring occurrence of abusive physical, sexual, and emotional events before the age of 18 (Dunne et al. 2009), Cronbach’s *α* 0.71 (7 items).

#### Perceived Parenting and Child Behavior

*Child maltreatment* (physical abuse, emotional abuse, and neglect) in the past month was assessed using a culturally adapted version of the ISPCAN Child Abuse Screening Tool (Meinck et al. 2018; Zolotor et al. 2009). For the child report, Cronbach’s *α* was 0.90, and for caregiver report, 0.79. *Parenting* approaches were measured by the Alabama Parenting Questionnaire (parent and child versions) (Frick 1991), an instrument widely used internationally, as well as in South Africa. As suggested by previous research with children of this age, and supported by exploratory factor analyses in this sample, we combined positive and involved parenting subscales. For the child report, Cronbach’s *α* were 0.92 for positive and involved parenting (16 items), 0.76 for poor monitoring (10 items), and 0.68 for inconsistent discipline (6 items). For the caregiver report, Cronbach’s *α* were 0.85 for positive and involved parenting (16 items), 0.72 for poor monitoring (10 items), and 0.55 for inconsistent discipline (6 items). Child externalizing behavior was measured using the rule-breaking and aggressive behavior scales of the Child Behavior Checklist 4–18 (Achenbach 1991). Cronbach’s *α* were 0.85 (child report) and 0.90 (caregiver report), 35 items each.

#### Sociodemographic Factors

Other participant characteristics used in the analyses were age, gender, caregiver employment, and child’s orphan status.

### Data Analysis

For the sessions attended by two observers, we examined inter-rater reliability of participant engagement measure. The intra-class correlation coefficients were 0.79 (95% CI 0.75; 0.81) for child engagement and 0.85 (95% CI 0.83; 0.87) for caregiver engagement. Given the high reliability, we used averages of two observations for analyses. To evaluate whether there were any systematic differences between caregiver and child attendance and engagement, we conducted *t* tests, adjusted for clustering. To examine changes in individual engagement over time, we used session number as a predictor of engagement in a multilevel model. To examine the level of overall attendance, we calculated percentages of participants attending each week out of the total number enrolled.

In the analyses of predictors, we used multilevel models to ensure that non-independence of data within clusters was appropriately taken into account (Hox et al. 2010). The analyses presented here include bivariate and multiple random-intercept models. We used restricted maximum likelihood estimation with Kenward-Roger correction, as recommended for samples with 10–20 clusters (McNeish 2017). As demonstrated elsewhere (Enders and Tofighi 2007), when the question primarily bears on variable relationships at the lowest level of the model (participants), group-mean centering of predictors provides an appropriate estimate of the relationship. Therefore, we used predictors centered around the cluster means. Thus, the regression coefficients represent pooled within-cluster relationships. The only group-level predictor (peri-urban or rural location) was grand-mean centered. To facilitate interpretation of continuous predictors other than age, they were standardized using pooled within-cluster standard deviations (available online). Participant engagement was standardized using the overall mean and standard deviation in the outcome. Because of the focus on participant-level variables, we used fixed slopes of predictors across clusters. A linear link function was used for all regression analyses presented here. As a sensitivity check, given the count nature of attendance outcomes, we also analyzed them using a negative-binomial link function with a robust sandwich estimator of variance and found the same pattern of results with one minor difference, discussed subsequently. All analyses were implemented in Stata/SE 14.2, except the inter-rater reliability calculations in R 3.3.0.

## Results

### Describing Participation in Sinovuyo Teen

Caregivers attended 7.1 group sessions on average (50% of all sessions) and children, 9 (64%). Thus, children attended more sessions than caregivers, although the difference did not reach statistical significance: *t*(540) = − 1.59, *p* = 0.12. Alternative caregivers could attend sessions, but it was not common, with 43 cases recorded across 270 families in 279 sessions. Twenty-five (9%) caregivers and 14 (5%) children did not attend any group sessions. As part of the program design, families were approached for home visits throughout the 14 weeks if they did not attend a session, unless they chose to drop out of the study. None of the families dropped out of the study during the intervention. Only four families received no visits or sessions. Given the small number, they were included in the main analyses.

Children’s mean engagement in sessions was 2.03 (SD = 0.49), 68% of maximum on the measure, and caregivers’, 2.40 (SD = 0.46), 80% of maximum. This difference between children and caregivers was significant: *t*(498) = 3.01, *p* < 0.01. Therefore, as a group, caregivers were rated as more actively engaged in the sessions they attended than children. For 25 (9.3%) caregivers and 17 (6.3%) children, engagement data were missing either because they did not attend any group sessions or were not rated by error. Consequently, the analyses of predictors for engagement have a smaller number of participants than the analyses for attendance. Attendance and engagement varied significantly across the clusters, with unconditional intra-class correlations of .24 for caregiver attendance, .06 for caregiver engagement, .12 for child attendance, and .15 for child engagement. Cluster’s rural or peri-urban status explained .30 of between-cluster variance for caregiver attendance, .31 for child attendance, .14 for caregiver engagement, and .22 for child engagement.

### Relation Between the Outcome Variables

Within families, there was a large correlation (.60) between the attendance of caregiver and child, and a medium correlation (.27) between the average engagement of caregiver and child. Given the size of the correlations, it was informative to analyze each of the outcomes separately to test their unique predictors.

### Participation over Time

**Fig. 1:**
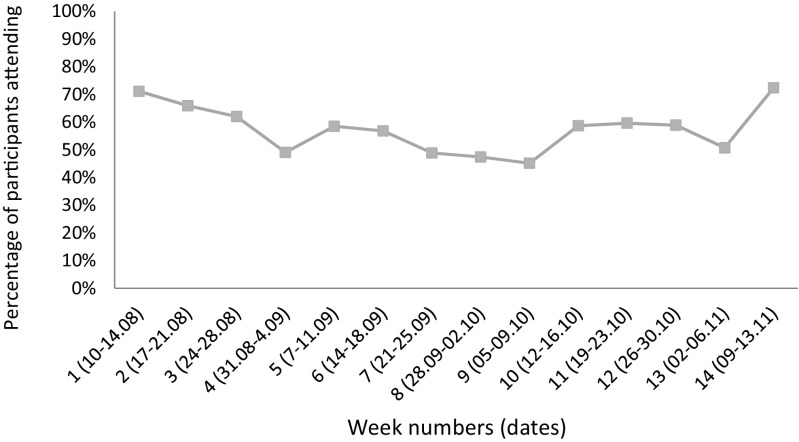
Percentage of participants attending group sessions each week (August–November 2015)

Figure 1 plots overall attendance across the 14 intervention weeks. While attendance did not consistently increase or decrease over time, there was an ongoing fluctuation. We observed that the dips in attendance approximately corresponded to the beginning of a new month when social grants were disbursed, and families traveled to receive them. Examining individual growth plots and longitudinal multilevel models, we found no linear trend for child engagement and a slight increase in caregiver engagement over time (*B* = 0.02, *p* < 0.001).

### Predictors of Attendance

Several predictors were significantly related to caregiver attendance in the multiple regression analysis (see Table 1). Peri-urban or rural residence, alcohol and substance use, positive and involved parenting, caregiver age, gender, and caregiver employment showed unique relationships to attendance in a model that included all predictors simultaneously. Specifically, caregivers in peri-urban areas attended on average 3.08 fewer sessions than caregivers in villages. Caregivers with one deviation higher alcohol and substance use reported 0.50 fewer sessions attended (*p* = 0.048). Attendance was 0.67 sessions higher among caregivers who reported one standard deviation higher levels of positive and involved parenting. Older caregivers had a slightly higher attendance, with one additional year of age predicting 0.05 more sessions attended. Male caregivers (*n* = 8) attended 3.37 sessions fewer. Caregivers who had a job at baseline attended an average of 3.08 fewer sessions, compared to caregivers who did not report being employed at baseline, controlling for other predictors.

**Table 1:** Predictors of caregiver attendance and engagement

Predictor	Caregiver session attendance (*n* = 270, *j* = 20)				Caregiver average engagement (*n* = 245, *j* = 20)			
	Bivariate regression		Multiple regression^1^		Bivariate regression		Multiple regression	
	Coef.	95% CI	Coef.	95% CI	Coef.	95% CI	Coef.	95% CI
Economic and educational barriers and resources								
SES (0–8 household items)	− 0.18	− 0.40; 0.04	− 0.11	− 0.35; 0.13	0.03	0.00; 0.05	0.02	− 0.01; 0.05
Overcrowded housing	0.20	− 0.91; 1.29	0.36	− 0.72; 1.44	− 0.01	− 0.15; 0.12	0.00	− 0.14; 0.14
Peri-urban residence	− 3.08*	− 5.68; − 0.47	− 3.08*	− 5.67; − 0.48	0.13	− 0.09; 0.34	0.14	− 0.08; 0.35
Caregiver completed primary school	− 0.89	− 1.97; 0.19	− 0.22	− 1.40; 0.96	0.14*	0.01; 0.27	0.14	− 0.01; 0.29
Social and health barriers and resources								
Caregiver depression	0.01	− 0.04; 0.05	− 0.01	− 0.05; 0.04	− 0.01*	− 0.01; 0.00	0.00	− 0.01; 0.00
Caregiver HIV-positive	0.14	− 0.94; 1.22	0.31	− 0.76; 1.37	− 0.11	− 0.24; 0.03	− 0.11	− 0.24; 0.03
Alcohol and substance use	− 0.85**	− 1.41; − 0.29	− 0.60*	− 1.19; − 0.01	− 0.08*	− 0.15; − 0.01	− 0.07	− 0.15; 0.01
Caregiver social support	− 0.01	− 0.06; 0.04	− 0.01	− 0.06; 0.04	0.00	− 0.01; 0.01	0.00	− 0.01; 0.00
Caregiver intimate partner violence exposure	− 0.16	− 0.56; 0.25	− 0.07	− 0.47; 0.34	− 0.05	− 0.10; 0.00	− 0.04	− 0.09; 0.01
Caregiver childhood maltreatment	0.00	− 0.35; 0.34	0.03	− 0.33; 0.40	0.00	− 0.05; 0.04	0.01	− 0.04; 0.05
Perceived parenting and child behavior								
Positive and involved parenting	0.06**	0.02; 0.10	0.06*	0.01; 0.11	0.00	0.00; 0.01	0.00	0.00; 0.01
Poor monitoring	− 0.03	− 0.09; 0.04	− 0.02	− 0.10; 0.06	0.00	− 0.01; 0.01	0.00	− 0.01; 0.01
Inconsistent discipline	0.05	− 0.06; 0.16	0.04	− 0.09; 0.16	0.00	− 0.01; 0.01	0.00	− 0.02; 0.01
Maltreatment	0.02	− 0.02; 0.07	0.02	− 0.03; 0.07	0.00	− 0.01; 0.00	0.00	− 0.01; 0.00
Child externalizing	− 0.02	− 0.06; 0.02	0.00	− 0.05; 0.05	0.00	0.00; 0.01	0.00	0.04; 0.01
Sociodemographic characteristics								
Caregiver age	0.05**	0.02; 0.08	0.05**	0.01; 0.09	0.00	0.00; 0.00	0.00	0.00; 0.01
Female caregiver	3.32*	0.58; 6.06	3.37*	0.67; 6.07	0.31	− 0.03; 0.65	0.32	− 0.02; 0.67
Child is an orphan	0.06	− 1.01; 1.12	− 0.16	− 1.22; 0.89	0.10	− 0.03; 0.23	0.09	− 0.04; 0.22
Caregiver is biological parent	− 0.07	− 1.07; 0.92	0.50	− 0.52; 1.52	− 0.06	− 0.19; 0.06	0.02	− 0.11; 0.15
Caregiver has a job	− 3.41**	− 5.55; − 1.26	− 3.08**	− 5.22; − 0.94	− 0.12	− 0.41; 0.16	− 0.10	− 0.39; 0.18
Random intercept			7.06***	6.03; 8.09			2.40***	2.32; 2.49

^1^Adjusted for all covariates shown; *p < 0.05, **p < 0.01, ***p < 0.001. All predictors besides residence were group-mean centered

Several unique predictors were significantly related to child attendance: peri-urban or rural residence, overcrowded household, alcohol and substance use, inconsistent parenting, and child age (see Table 2). Similarly to caregivers, young people in peri-urban areas attended on average 2.29 fewer sessions than in the villages. On the other hand, children in overcrowded households attended 1.21 more sessions. Children with one deviation higher reported alcohol and substance use attended 0.58 fewer sessions. Older children attended fewer sessions, with one additional year of age predicting 0.39 fewer sessions attended. Finally, children who reported higher inconsistent parenting also attended more sessions—this finding, however, was impacted by interactions with other variables as there was no bivariate relation between the two variables.

**Table 2:** Predictors of child attendance and engagement

Predictor	Child session attendance (*n* = 270, *j* = 20)				Child average engagement (*n* = 253, *j* = 20)			
	Bivariate regression		Multiple regression^2 ^		Bivariate regression		Multiple regression	
	Coef.	95% CI	Coef.	95% CI	Coef.	95% CI	Coef.	95% CI
Economic and educational barriers and resources								
SES (0–8 household items)	− 0.04	− 0.27; 0.19	− 0.03	− 0.24; 0.19	− 0.01	− 0.03; 0.02	0.00	− 0.03; 0.02
Overcrowded housing	1.64**	0.47; 2.81	1.21*	0.10; 2.31	0.03	− 0.11; 0.16	0.07	− 0.07; 0.20
Peri-urban residence	− 2.27*	− 4.39; − 0.16	− 2.29*	− 4.39; − 0.18	0.23	− 0.03; 0.50	0.24	− 0.02; 0.51
Social and health barriers and resources								
Child depression	0.05	− 0.13; 0.23	0.02	− 0.17; 0.21	0.01	− 0.01; 0.03	0.01	− 0.01; 0.04
Child HIV-positive	1.31*	0.12; 2.50	0.77	− 0.36; 1.89	0.02	− 0.11; 0.15	0.04	− 0.10; 0.18
Alcohol and substance use	− 1.14***	− 1.62; − 0.66	− 0.57*	− 1.10; − 0.03	0.02	− 0.04; 0.09	0.00	− 0.08; 0.08
Perceived parenting and child behavior								
Positive and involved parenting	0.04*	0.01; 0.08	− 0.01	− 0.06; 0.03	0.00	0.00; 0.01	0.00	0.00; 0.01
Poor monitoring	− 0.11**	− 0.17; − 0.04	− 0.07	− 0.15; 0.01	0.01	0.00; 0.02	0.00	− 0.01; 0.01
Inconsistent discipline	0.06	− 0.05; 0.17	0.16*	0.03; 0.29	0.02*	0.01; 0.03	0.02*	0.00; 0.03
Maltreatment	− 0.03	− 0.06; 0.00	− 0.01	− 0.04; 0.02	0.00	0.00; 0.00	0.00	0.00; 0.00
Child externalizing	− 0.13***	− 0.19; − 0.07	− 0.06	− 0.13; 0.02	0.00	0.00; 0.01	0.00	− 0.01; 0.01
Sociodemographic characteristics								
Child age	− 0.63***	− 0.84; − 0.42	− 0.39**	− 0.63; − 0.15	0.03*	0.01; 0.06	0.03*	0.00; 0.06
Female child	0.32	− 0.74; 1.38	− 0.03	− 1.04; 0.98	0.00	− 0.12; 0.12	0.00	− 0.13; 0.12
Child is an orphan	0.57	− 0.57; 1.71	0.86	− 0.22; 1.94	0.04	− 0.09; 0.17	0.07	− 0.06; 0.21
Caregiver is biological parent	− 0.37	− 1.44; 0.70	0.09	− 0.92; 1.09	0.04	− 0.08; 0.16	0.01	− 0.11; 0.14
Random intercept			8.98***	8.16; 9.81			2.04***	1.94; 2.13

^2^Adjusted for all covariates shown; *p < 0.05, **p < 0.01, ***p < 0.001. All predictors besides residence were group-mean centered

In the sensitivity analyses (available online), one result differed in terms of statistical significance. Using negative-binomial models, child alcohol and substance use was not a statistically significant predictor (*p* = 0.127) of attendance.

### Predictors of Engagement

None of the predictors were significantly related to caregiver engagement in multiple regression (see Table 1). Examining the predictors of child engagement, two predictors showed unique relations to engagement: inconsistent parenting and child age (see Table 2). Children reporting one standard deviation more inconsistent parenting had 0.17 standard deviations higher engagement. Older children also had slightly higher engagement, with an additional year of age predicting 0.07 standard deviations higher engagement. VIF values ranged between 1.04 and 2.10, suggesting multicollinearity was not a concern.

## Discussion

Delivering evidence-informed services can only be beneficial if families participate in them. In this study, we explored factors that influenced attendance and engagement for caregivers and children enrolled in a parenting program in a disadvantaged area of South Africa. The study did not yield evidence that family disadvantage was related to levels of attendance and engagement. This may be due to the program design including efforts to reduce known barriers to engagement, for instance, by providing transport.

Overall, the session attendance rates in this study are somewhat lower than the average rates of around 72% reported in parenting program studies in HICs (Chacko et al. 2016). Other studies in LMICs report even higher attendance rates, such as 81.2% for caregivers and children 7–15 years old in a 12-session program among Burmese migrant and displaced population in Thailand (Annan et al. 2017). One reason for this difference may be the provision of home visits in Sinovuyo Teen for all participants who missed a session, reducing the incentive to attend group sessions. There may be a trade-off between reaching participants with home visits and their additional costs.

As in many previous studies, individual socio-economic status did not predict participation. However, participants in rural clusters attended more sessions than those in peri-urban areas, possibly due to fewer alternative services or leisure opportunities in the villages. South Africa’s rural areas continue to have lower levels of public services, income, and government grants than peri-urban or urban locations (Coovadia et al. 2009). Moreover, we found higher attendance rates among children from overcrowded households. One interpretation is a higher perceived need for support by youth in overcrowded homes. The sessions may have also provided a break from a crowded home.

Overall, these are encouraging findings that suggest parenting programs can successfully reach vulnerable families. However, we found that both caregivers and children with higher rates of alcohol and substance use have lower attendance, although the difference for children did not reach statistical significance in the sensitivity analyses. Thus, some social and health barriers, such as alcohol and substance use, can still hinder participation and need to be investigated further.

Similar to much of the previous research, mental health and social resources, as well as parenting and child behavior, were generally not related to participation. However, several parenting dimensions did appear as significant predictors. Higher positive and involved parenting predicted higher caregiver attendance, and more inconsistent parenting predicted more child attendance and higher engagement.

In line with other parenting studies, this sample included mostly female caregivers, and the few male caregivers attended much less frequently. This likely has to do with the social norms of women bearing the responsibility for childcare. Engaging men in parenting interventions requires a conscious effort in program design and delivery, such as drawing on specific motivations for fathers (Siu et al. 2017). In addition, children attended more sessions than caregivers, but had a lower average engagement in sessions, perhaps due to the cultural norms mandating that children show respect to elders. Children’s lower engagement may also be related to the pedagogical style common in schools, with children as passive listeners.

Consistent with previous research, time and logistics emerged as a major barrier to attendance. Caregivers who were employed had lower attendance—likely because the sessions took place on workday afternoons. It was not feasible due to safety issues to conduct sessions in the evening, which could help working caregivers. In the follow-up questionnaire, both children and caregivers cited other commitments as the most common reason for not attending: community events, such as church group meetings and funerals, family obligations, such as housework, and school commitments for children. In addition, sickness was an oft-cited reason for not attending. Monthly drops in attendance seemed to coincide with the time when participants traveled to obtain their monthly government grants and then shop for food and other necessities. While this has not been highlighted in previous literature, flexible scheduling to accommodate community events may be beneficial in rural settings.

The study has several limitations. First, the limited statistical power requires replication of the results in other studies. Future intervention studies may benefit from incorporating pre-planned analyses of program enrolment, attendance, and engagement into their trial protocols with power calculations. Second, the findings may not be generalizable to other settings. For example, three out of the four peri-urban clusters included in the intervention were part of one township area. However, this area was very populous, with over 18,000 residents. Third, we were only able to examine variation among study participants, and do not know if the most disadvantaged families enrolled in the trial at similar rates. Future research in LMICs also needs to examine programmatic factors, such as recruitment strategies and relationship with the facilitators, and their interaction with attendance. Examining participant perceptions of barriers to treatment and caregiver causal attributions of children’s behaviors in LMICs can inform interventions to boost engagement, such as motivational interviewing.

This study contributes to the scarce literature on evaluating the delivery of family interventions in LMIC settings by examining a range of relevant predictors of attendance and engagement. This is also one of the few studies to examine child or adolescent attendance. Based on this study and other recent research, it appears that parenting support programs can reach and engage very vulnerable families. Moreover, the most vulnerable families in LMICs may be especially receptive to these programs. These findings have important implications for programming and policy. The next vital question is whether these levels of participation can be maintained when the programs are disseminated more widely in service settings.

## Electronic Supplementary Material

ESM 1 (DOCX 58 kb)
