# Supplementary material for: What Affects Attendance and Engagement in a Parenting Program in South Africa?
Source: Prev Sci. 2018 Aug 18;19(7):977–86. doi: 10.1007/s11121-018-0941-2 (PMC6182387; doi:10.1007/s11121-018-0941-2)
Supplement: Supplementary file 1 — (DOCX 58 kb) [file 11121_2018_941_MOESM1_ESM.docx]

**Supplementary Material**

Appendix Table 1. *Baseline Sample Characteristics*

| Variable | Intervention arm |
| --- | --- |
| Xhosa-speaking (African) | 100% |
| Rural village  Peri-urban township | 83%  17% |
| Caregiver age, mean(*SD*) | 49 years (15.2) |
| Caregiver gender | 97% female |
| People living in the household, mean(*SD*) | 5.4 (2.3) |
| No one working in the household | 66% |
| At least 2 days in the past week with not enough food at home | 67% |
| HIV prevalence for children | 23% |
| HIV prevalence for caregivers | 26% |

Appendix Table 2*. Baseline Distribution of the Predictors of Caregiver Attendance and Engagement*

| Predictor | Mean (SD) | Observed range | Possible range |
| --- | --- | --- | --- |
| *Economic and educational barriers and resources* | | |  |
| SES (0-8 household items) | 3.86 (2.23) | 0-8 | 0-8 |
| Overcrowded housing | 0.27 (0.45) | 0-1 | 0-1 |
| Peri-urban township | 0.17 (0.38) | 0-1 | 0-1 |
| Caregiver completed primary school | 0.74 (0.44) | 0-1 | 0-1 |
| *Social and health barriers and resources* | | |  |
| Caregiver depression | 23.13 (11.78) | 0-54 | 0-57 |
| Caregiver HIV-positive | 0.26 (0.44) | 0-1 | 0-1 |
| Alcohol & substance use | 0.46 (0.88) | 0-3 | 0-4 |
| Caregiver social support | 26.00 (9.82) | 0-38 | 0-38 |
| Caregiver intimate partner violence exposure | 0.44 (1.17) | 0-6 | 0-6 |
| Caregiver childhood maltreatment | 1.06 (1.41) | 0-7 | 0-7 |
| *Perceived parenting and child behaviour* | | |  |
| Positive and involved parenting | 36.54 (11.10) | 6-62 | 0-64 |
| Poor monitoring | 13.85 (7.54) | 0-34 | 0-40 |
| Inconsistent discipline | 8.89 (4.37) | 0-20 | 0-24 |
| Maltreatment | 8.94 (10.65) | 0-76 | 0-136 |
| Child externalizing | 18.94 (12.01) | 0-56 | 0-70 |
| *Socio demographic characteristics* | | | |
| Caregiver age | 48.79 (15.20) | 18-92 |  |
| Female caregiver | 0.97 (0.17) | 0-1 | 0-1 |
| Child is an orphan | 0.28 (0.45) | 0-1 | 0-1 |
| Caregiver is biological parent | 0.37 (0.48) | 0-1 | 0-1 |
| Caregiver has a job | 0.05 (0.22) | 0-1 | 0-1 |

Appendix Table 3. *Baseline Distribution of the Predictors of Child Attendance and Engagement*

| Predictor | Mean (SD) | Observed range | Possible range |
| --- | --- | --- | --- |
| *Economic and educational barriers and resources* | | | |
| SES (0-8 household items) | 3.86 (2.23) | 0-8 | 0-8 |
| Overcrowded housing | 0.27 (0.45) | 0-1 | 0-1 |
| Peri-urban township | 0.17 (0.38) | 0-1 | 0-1 |
| *Social and health barriers and resources* | | | |
| Child depression | 16.65 (2.84) | 7-20 | 0-20 |
| Child HIV-positive | 0.23 (0.42) | 0-1 | 0-1 |
| Alcohol & substance use | 0.60 (1.09) | 0-6 | 0-6 |
| *Perceived parenting and child behaviour* | | | |
| Positive and involved parenting | 32.23 (15.02) | 0-64 | 0-64 |
| Poor monitoring | 14.16 (7.87) | 0-40 | 0-40 |
| Inconsistent discipline | 7.40 (4.91) | 0-20 | 0-24 |
| Maltreatment | 12.40 (17.64) | 0-120 | 0-144 |
| Child externalizing | 14.24 (8.60) | 0-46 | 0-70 |
| *Sociodemographic characteristics* | | | |
| Child age | 13.74 (2.33) | 10-18 | 10-18 |
| Female child | 0.42 (0.49) | 0-1 | 0-1 |
| Child is an orphan | 0.28 (0.45) | 0-1 | 0-1 |
| Caregiver is biological parent | 0.37 (0.48) | 0-1 | 0-1 |

Appendix Table 4. *Range of Group Means for Attendance and Engagement*

| Variable | Cluster means |
| --- | --- |
| Attendance child | 4.88-12.88 |
| Attendance caregiver | 3.63-12.00 |
| Engagement child | 1.71-2.54 |
| Engagement caregiver | 2.08-2.70 |

| Table 5. *Predictors of Caregiver Attendance and Engagement (Standardized Predictors)* | | | | | | | | |
| --- | --- | --- | --- | --- | --- | --- | --- | --- |
|  | Caregiver session attendance (*n*=270, *j*=20) | | | | Caregiver average engagement‡ (*n*=245, *j*=20) | | | |
| Predictor | Bivariate regression | | Multiple regression^[[1]](#footnote-1)^ | | Bivariate regression | | Multiple regression | |
|  | Coef. | 95 % CI | Coef. | 95 % CI | Coef. | 95 % CI | Coef. | 95 % CI |
| Economic and educational barriers and resources | | | | | | | | |
| SES (0-8 household items) † | -0.39 | -0.87; 0.08 | -0.24 | -0.76; 0.29 | 0.12 | 0.00; 0.25 | 0.10 | -0.05; 0.24 |
| Overcrowded housing | 0.20 | -0.91; 1.29 | 0.36 | -0.72; 1.44 | -0.03 | -0.32; 0.27 | 0.00 | -0.30; 0.29 |
| Peri-urban residence | -3.08* | -5.68; -0.47 | -3.08* | -5.67; -0.48 | 0.27 | -0.19; 0.74 | 0.29 | -0.18; 0.76 |
| Caregiver completed primary school | -0.89 | -1.97; 0.19 | -0.22 | -1.40; 0.96 | 0.31* | 0.03; 0.59 | 0.31 | 0.02; 0.27 |
| Social and health barriers and resources | | | | | | | | |
| Caregiver depression† | 0.07 | -0.41; 0.55 | -0.07 | -0.59; 0.44 | -0.15* | -0.27; -0.02 | -0.07 | -0.21; 0.07 |
| Caregiver HIV-positive | 0.14 | -0.94; 1.22 | 0.31 | -0.76; 1.37 | -0.23 | -0.52; 0.06 | -0.23 | -0.52; 0.07 |
| Alcohol & substance use† | -0.72** | -1.18; -0.25 | -0.50* | -1.00; 0.00 | -0.14* | -0.27; -0.01 | -0.13 | -0.26; 0.01 |
| Caregiver social support† | -0.07 | -0.55; 0.40 | -0.05 | -0.55; 0.44 | 0.00 | -0.13; 0.13 | -0.05 | -0.19; 0.09 |
| Caregiver intimate partner violence exposure† | -0.19 | -0.66; 0.29 | -0.08 | -0.56; 0.41 | -0.13 | -0.26; 0.00 | -0.10 | -0.24; 0.03 |
| Caregiver childhood maltreatment † | -0.01 | -0.48; 0.47 | 0.04 | -0.46; 0.55 | -0.02 | -0.14; 0.11 | 0.02 | -0.12; 0.16 |
| *Perceived parenting and child behaviour* | | | | | | | | |
| Positive and involved parenting † | 0.70** | 0.23; 1.17 | 0.67* | 0.15; 1.19 | 0.05 | -0.07; 0.18 | 0.04 | -0.10; 0.18 |
| Poor monitoring † | -0.19 | -0.67; 0.29 | -0.14 | -0.72; 0.44 | -0.03 | -0.16; 0.10 | -0.07 | -0.23; 0.10 |
| Inconsistent discipline † | 0.21 | -0.27; 0.69 | 0.16 | -0.39; 0.71 | -0.01 | -0.14; 0.11 | -0.02 | -0.17; 0.13 |
| Maltreatment † | 0.24 | -0.24; 0.71 | 0.22 | -0.29; 0.72 | -0.08 | -0.20; 0.05 | -0.08 | -0.22; 0.06 |
| Child externalizing † | -0.25 | -0.72; 0.23 | 0.04 | -0.53; 0.61 | 0.02 | -0.10; 0.15 | 0.12 | -0.04; 0.28 |
| *Sociodemographic characteristics* | | | | | | | | |
| Caregiver age | 0.05** | 0.02; 0.08 | 0.05** | 0.01; 0.09 | 0.00 | -0.01; 0.01 | 0.00 | -0.01; 0.01 |
| Female caregiver | 3.32* | 0.58; 6.06 | 3.37* | 0.67; 6.07 | 0.68 | -0.07; 1.42 | 0.70 | -0.05; 1.45 |
| Child is an orphan | 0.06 | -1.01; 1.12 | -0.16 | -1.21; 0.82 | 0.22 | -0.06; 0.50 | 0.20 | -0.09; 0.48 |
| Caregiver is biological parent | -0.07 | -1.07; 0.92 | 0.50 | -0.52; 1.52 | -0.14 | -0.40; 0.13 | 0.05 | -0.24; 0.33 |
| Caregiver has a job | -3.41** | -5.55; -1.26 | -3.08** | -5.22; -0.94 | -0.27 | -0.89; 0.35 | -0.23 | -0.84; 0.39 |
| *Random Intercept* |  |  | 7.06*** | 6.03; 8.09 |  |  | 0.02 | -0.17; 0.20 |

| Table 6. *Predictors of Child Attendance and Engagement (Standardized Predictors)* | | | | | | | | |
| --- | --- | --- | --- | --- | --- | --- | --- | --- |
|  | Child session attendance (*n*=270, *j*=20) | | | | Child average engagement‡ (*n*=253, *j*=20) | | | |
| Predictor | Bivariate regression | | Multiple regression^[[2]](#footnote-2)^ | | Bivariate regression | | Multiple regression | |
|  | Coef. | 95 % CI | Coef. | 95 % CI | Coef. | 95 % CI | Coef. | 95 % CI |
| *Economic and educational barriers and resources* | | | | | | | | |
| SES (0-8 household items) † | -0.09 | -0.61; 0.42 | -0.06 | -0.53; 0.42 | -0.03 | -0.15; 0.09 | -0.02 | -0.14; 0.10 |
| Overcrowded housing | 1.64** | 0.47; 2.81 | 1.21* | 0.10; 2.31 | 0.05 | -0.22; 0.33 | 0.14 | -0.14; 0.41 |
| Peri-urban residence † | -2.27* | -4.39; -0.16 | -2.29* | -4.39; -0.18 | 0.48 | -0.06; 1.03 | 0.50 | -0.04; 1.04 |
| *Social and health barriers and resources* | | | | | | | | |
| Child depression † | 0.14 | -0.37; 0.65 | 0.05 | -0.47; 0.57 | 0.07 | -0.05; 0.19 | 0.07 | -0.06; 0.20 |
| Child HIV-positive | 1.31* | 0.13; 2.50 | 0.77 | -0.36; 1.89 | 0.04 | -0.24; 0.31 | 0.08 | -0.20; 0.37 |
| Alcohol & substance use † | -1.17*** | -1.66; -0.68 | -0.58* | -1.13; -0.03 | 0.05 | -0.09; 0.18 | 0.00 | -0.16; 0.16 |
| *Perceived parenting and child behaviour* | | | | | | | | |
| Positive and involved parenting † | 0.58* | 0.07; 1.09 | -0.21 | -0.80; 0.37 | 0.08 | -0.03; 0.20 | 0.03 | -0.11; 0.18 |
| Poor monitoring † | -0.78** | -1.29; -0.28 | -0.49 | -1.07; 0.08 | 0.12 | 0.00; 0.23 | 0.00 | -0.15; 0.16 |
| Inconsistent discipline † | 0.26 | -0.25; 0.78 | 0.75* | 0.16; 1.35 | 0.18* | 0.06; 0.30 | 0.17* | 0.02; 0.33 |
| Maltreatment † | -0.53 | -1.04; 0.02 | -0.18 | -0.70; 0.34 | 0.03 | -0.08; 0.15 | 0.00 | -0.13; 0.13 |
| Child externalizing † | -1.05*** | -1.54; -0.55 | -0.46 | -1.06; 0.13 | 0.04 | -0.07; 0.16 | -0.01 | -0.16; 0.15 |
| *Sociodemographic characteristics* | | | | | | | | |
| Child age | -0.63*** | -0.84; -0.42 | -0.39** | -0.63; -0.15 | 0.06* | 0.01; 0.12 | 0.07* | 0.01; 0.13 |
| Female child | 0.32 | -0.74; 1.38 | -0.03 | -1.04; 0.98 | 0.00 | -0.25; 0.24 | 0.00 | -0.26; 0.25 |
| Child is an orphan | 0.57 | -0.57; 1.72 | 0.86 | -0.22; 1.94 | 0.08 | -0.19; 0.34 | 0.15 | -0.12; 0.43 |
| Caregiver is biological parent | -0.37 | -1.44; 0.70 | 0.09 | -0.92; 1.09 | 0.07 | -0.17; 0.32 | 0.02 | -0.23; 0.28 |
| *Random Intercept* |  |  | 8.98*** | 8.16; 9.81 |  |  | 0.01 | 0.20; 0.22 |

Appendix Table 7. *Sensitivity Analysis for Predictors of Caregiver Attendance (Negative-Binomial Model)*

|  | Caregiver session attendance (*n*=270, *j*=20) | | | | |
| --- | --- | --- | --- | --- | --- |
| Predictor | Bivariate regression | | Multiple regression^[[3]](#footnote-3)^ | | |
|  | IRR^[[4]](#footnote-4)^ | 95 % CI | IRR | 95 % CI | |
| *Economic and educational barriers and resources* | | | | |  |
| SES (0-8 household items) | 0.98 | 0.95; 1.01 | 0.98 | 0.94, 1.02 | |
| Overcrowded housing | 1.02 | 0.82; 1.26 | 1.04 | 0.86; 1.27 | |
| Peri-urban township | 0.61*** | 0.50; 0.75 | 0.59*** | 0.47; 0.75 | |
| Caregiver completed primary school | 0.86 | 0.72; 1.03 | 0.97 | 0.83; 1.13 | |
| *Social and health barriers and resources* | | | | |  |
| Caregiver depression | 1.00 | 0.99; 1.01 | 1.00 | 0.99; 1.00 | |
| Caregiver HIV-positive | 1.00 | 0.81; 1.23 | 1.04 | 0.87; 1.25 | |
| Alcohol & substance use | 0.86* | 0.77; 0.97 | 0.89* | 0.80; 0.98 | |
| Caregiver social support | 1.00 | 0.99; 1.00 | 1.00 | 0.99; 1.01 | |
| Caregiver intimate partner violence exposure | 0.98 | 0.91; 1.05 | 1.00 | 0.93; 1.08 | |
| Caregiver childhood maltreatment | 1.00 | 0.95; 1.05 | 1.01 | 0.96; 1.07 | |
| *Perceived parenting and child behaviour* | | | | |  |
| Positive and involved parenting | 1.01** | 1.00; 1.02 | 1.01* | 1.00; 1.02 | |
| Poor monitoring | 1.00 | 0.99; 1.01 | 1.00 | 0.99; 1.01 | |
| Inconsistent discipline | 1.01 | 0.99; 1.02 | 1.01 | 1.00; 1.02 | |
| Maltreatment | 1.00 | 1.00; 1.01 | 1.01 | 1.00; 1.02 | |
| Child externalizing | 1.00 | 0.99; 1.00 | 1.00 | 0.99; 1.01 | |
| *Sociodemographic characteristics* | | | | |  |
| Caregiver age | 1.01* | 1.00; 1.02 | 1.01* | 1.00; 1.02 | |
| Female caregiver | 1.88* | 1.14; 3.09 | 1.85* | 1.07; 3.18 | |
| Child is an orphan | 1.02 | 0.84; 1.24 | 0.98 | 0.83; 1.16 | |
| Caregiver is biological parent | 0.96 | 0.79; 1.17 | 1.07 | 0.92; 1.25 | |
| Caregiver has a job | 0.53** | 0.35; 0.80 | 0.57** | 0.34; 0.85 | |
| *Intercept* |  |  | 6.53*** | 5.67; 7.53 | |

Appendix Table 8*. Sensitivity Analysis for Predictors of Child Attendance (Negative-Binomial Model)*

|  | Child session attendance (n=270, j=20) | | | | |
| --- | --- | --- | --- | --- | --- |
| Predictor | Bivariate regression | | Multiple regression | | |
|  | IRR | 95 % CI | IRR | 95 % CI | |
| *Economic and educational barriers and resources* | | | | |  |
| SES (0-8 household items) | 1.00 | 0.98; 1.01 | 1.00 | 0.98; 1.01 | |
| Overcrowded housing | 1.21** | 1.08; 1.35 | 1.16* | 1.03; 1.31 | |
| Peri-urban township | 0.76* | 0.60; 0.96 | 0.74* | 0.57; 0.96 | |
| *Social and health barriers and resources* | | | | |  |
| Child depression | 1.00 | 0.98; 1.03 | 1.00 | 0.98; 1.03 | |
| Child HIV-positive | 1.15** | 1.06; 1.25 | 1.07 | 0.99; 1.17 | |
| Alcohol & substance use | 0.84** | 0.76; 0.93 | 0.91 | 0.80; 1.03 | |
| *Perceived parenting and child behaviour* | | | | |  |
| Positive and involved parenting | 1.00* | 1.00; 1.01 | 1.00 | 0.99; 1.00 | |
| Poor monitoring | 0.99** | 0.98; 0.99 | 0.99 | 0.98; 1.00 | |
| Inconsistent discipline | 1.01 | 0.99; 1.02 | 1.02* | 1.00; 1.04 | |
| Maltreatment | 1.00 | 0.99; 1.00 | 1.00 | 0.99; 1.00 | |
| Child externalizing | 0.98 | 0.98; 0.99 | 0.99 | 0.98; 1.00 | |
| *Sociodemographic characteristics* | | | | |  |
| Child age | 0.93*** | 0.89; 0.97 | 0.95* | 0.92; 0.99 | |
| Female child | 1.03 | 0.86; 1.24 | 0.99 | 0.83; 1.17 | |
| Child is an orphan | 1.07 | 0.92; 1.25 | 1.12 | 0.98; 1.30 | |
| Caregiver is biological parent | 0.95 | 0.84; 1.08 | 1.01 | 0.90; 1.12 | |
| *Intercept* |  |  | 8.63*** | 7.85; 9.49 | |

1. Adjusted for all covariates shown; † Predictor was standardized using group mean and pooled within-group standard deviations;

   ‡ Outcome was standardized using sample mean and standard deviation; **p*<0.05, ***p*<0.01, ****p*<0.001 [↑](#footnote-ref-1)
2. Adjusted for all covariates shown; † Predictor was standardized using group mean and pooled within-group standard deviations;

   ‡ Outcome was standardized using sample mean and standard deviation; **p*<0.05, ***p*<0.01, ****p*<0.001 [↑](#footnote-ref-2)
3. Adjusted for all covariates shown

   **p*<0.05, ***p*<0.01, ****p*<0.001 [↑](#footnote-ref-3)
4. Incidence rate ratio (IRR), IRR=1.00 no difference, IRR<1.00 lower attendance, IRR>1.00 higher attendance [↑](#footnote-ref-4)
